# Probabilistic health risk assessment of respirable dust exposure among stone carvers in Thailand using average daily dose and Monte Carlo simulation

**DOI:** 10.1371/journal.pone.0359706

**Published:** 2026-10-05

**Authors:** Arroon Ketsakorn, Ratchapong Chaiyadej

**Affiliations:** Faculty of Public Health, Thammasat University, Pathum Thani, Thailand; National Autonomous University of Mexico Institute of Geophysics: Universidad Nacional Autonoma de Mexico Instituto de Geofisica, MEXICO

## Abstract

Respirable dust (RD) exposure represents a significant occupational health concern in stone processing settings, particularly among stone carvers who are at elevated risk due to high levels of dust generation. Prolonged exposure to RD is associated with serious adverse health outcomes, including lung cancer. This study aimed to evaluate the probabilistic risk associated with RD exposure using Monte Carlo simulation and to estimate the Incremental Lifetime Cancer Risk (ILCR) among stone carvers. A cross-sectional study was conducted across five occupational categories: mining, cutting, splitting, knocking, and stone carving. Personal air sampling was performed in accordance with the NIOSH 7601 method to determine time-weighted average (TWA) RD concentrations. ILCR was estimated using Monte Carlo simulation with 10,000 iterations, incorporating exposure parameters and health risk assessment models based on U.S. EPA guidelines. The mean RD concentrations did not exceed either the Thai occupational exposure limit (OEL) or the threshold limit value (TLV) in any of the five occupational categories, with the highest level observed among stone carvers (0.051 mg/m^3^). Although ILCR values for all groups were within the acceptable threshold of 1.00 × 10 ^−6^, the highest mean ILCR was identified in the stone carving group (3.325 × 10 ^−7^). These findings suggest that, despite being within acceptable limits, workers may still face potential health risks from RD exposure. The results underscore the need for strengthened regulatory enforcement and comprehensive control strategies, including engineering controls, respiratory protection, and targeted health education, to mitigate exposure and prevent silica-related diseases in this high-risk population.

## Introduction

Respirable dust (RD) is an important occupational hazard that can adversely affect the human respiratory system. Prolonged inhalation of RD may result in respiratory irritation, pulmonary inflammation, and reduced lung function. The severity of these health effects depends on the dust composition, particle size, airborne concentration, and duration of exposure. Therefore, exposure to RD generated during stone-processing activities represents an important occupational health concern among stone carvers. The body’s defense mechanisms trigger alveolar macrophages to engulf and attempt to remove the deposited particles; however, this process induces fibrotic changes in the lung tissue (pulmonary fibrosis). Consequently, the efficiency of gas exchange in the alveoli is reduced, impairing oxygen transfer into the bloodstream. Affected individuals may experience symptoms such as shortness of breath, fatigue, chronic cough, and difficulty breathing, which can ultimately lead to death [1]. Although respirable dust (RD) as a general category has not been classified by the International Agency for Research on Cancer (IARC) as a Group 1 carcinogen, prolonged and repeated exposure to RD may still pose significant occupational health risks, including respiratory irritation, pulmonary inflammation, and impaired lung function [2].

The World Health Organization (WHO) and the International Labour Organization (ILO) have emphasized the importance of preventing and controlling occupational exposure to airborne and respirable dust (RD). Prolonged and repeated inhalation of RD may cause respiratory irritation, pulmonary inflammation, pneumoconiosis, reduced pulmonary function, and other occupational respiratory diseases [3,4]. The health risks associated with RD exposure depend on the physical and chemical characteristics of the dust, its airborne concentration, and the duration and frequency of exposure. In Thailand, occupational exposure to RD remains an important issue for workers in industries involving mining, construction, stone processing, manufacturing, and other dust-generating activities. Therefore, effective occupational health program should include exposure assessment, workplace dust monitoring, engineering and administrative controls, appropriate respiratory protection, worker training, and health surveillance. Medical surveillance may include respiratory symptom assessment, pulmonary function testing, and chest radiography when appropriate to the worker’s exposure history and clinical risk. In addition, the Department of Labour Protection and Welfare has issued regulations specifying occupational exposure limits for respirable dust. The permissible exposure limit for respirable dust, expressed as an 8-hour time-weighted average (TWA), must not exceed 5 mg/m^3^ [5]. These regulatory measures are intended to ensure that employers implement appropriate monitoring and preventive strategies to reduce respirable dust exposure in the workplace.

Respirable dust (RD) consists of fine airborne particles that can penetrate deeply into the lungs and reach the alveolar region. When RD contains crystalline silica, prolonged and repeated inhalation may lead to silicosis, a chronic fibrotic lung disease associated with persistent pulmonary inflammation and progressive scarring of lung tissue. The risk of developing silicosis depends on the crystalline silica content of the dust, airborne dust concentration, particle size, duration of exposure, and cumulative exposure. Therefore, occupational exposure to RD containing crystalline silica is an important risk factor for silicosis. The public health significance of this exposure is reflected in the surveillance data reported in Thailand. According to the Health Data Center (HDC), a centralized national health database maintained by the Ministry of Public Health, surveillance data on silicosis have been systematically collected since 2013. Between 2013 and 2022, a total of 1,779 cumulative silicosis cases were reported nationwide [6]. Nakhon Ratchasima Province recorded the highest cumulative number of cases, with 538 cases, accounting for approximately 30% of all reported cases in the country. Since 2016, Nakhon Ratchasima has consistently ranked among the top three provinces with the highest reported incidence rates of silicosis per 100,000 population. In 2022, the province reported 66 new cases, corresponding to an incidence rate of 3.44 per 100,000 population, approximately 6.7 times higher than the national average. This placed Nakhon Ratchasima second among the provinces in terms of incidence rate, after Saraburi Province, which reported the highest rate at 4.38 per 100,000 population. These findings highlight the importance of assessing and controlling occupational exposure to RD, particularly RD containing crystalline silica, together with appropriate exposure monitoring and health-surveillance program. Sikhio District in Nakhon Ratchasima Province has been identified as a sandstone resource area dating approximately 140–65 million years ago. The sandstone found in this region is highly resistant to weathering, which has supported the development of local wisdom in utilizing available raw materials for stone processing and carving. The sandstone found in this region is highly resistant to weathering, which has supported the development of local knowledge in utilizing available raw materials for stone processing and carving. These materials are commonly crafted into a variety of products, including decorative wall stones, paving stones, and Buddha statues, and over time, stone carving has become a primary occupation and a significant source of income for local communities. In Thailand, such stone processing and carving activities represent an important economic sector, particularly within local and small-scale enterprises. However, occupational health and safety measures in these settings are often inadequate, leading to elevated exposure risks among workers. Despite the establishment of occupational exposure limits (OELs) and threshold limit values (TLVs) to control RD containing crystalline silica exposure, several studies have reported that actual exposure levels frequently exceed these standards, especially in developing and informal sectors [7,8].

Traditional health risk assessments of RD exposure have typically relied on deterministic approaches, which may not adequately capture variability and uncertainty in exposure conditions and individual susceptibility [9]. In contrast, probabilistic risk assessment methods, such as Monte Carlo simulation, provide a more robust framework by incorporating distributions of input parameters and generating a range of possible risk outcomes. This approach allows for a more comprehensive evaluation of health risks, particularly in complex occupational environments. One of the key advantages of Monte Carlo simulation is its ability to explicitly account for variability and uncertainty in model inputs, thereby producing probabilistic risk estimates that better reflect real-world exposure scenarios. It also enables sensitivity analysis to identify the most influential parameters affecting risk outcomes, which is valuable for targeted risk management. Furthermore, the method supports decision-making by providing confidence intervals and risk distributions rather than single-point estimates [10,11]. However, Monte Carlo simulation also has several limitations. The accuracy of the results depends heavily on the quality and appropriateness of the input data and assumed probability distributions. In cases where empirical data are limited, assumptions may introduce bias or uncertainty into the model outputs. Additionally, the method can be computationally intensive and may require specialized expertise to implement and interpret correctly [12,13]. Despite these limitations, Monte Carlo simulation remains a powerful tool for enhancing the robustness of health risk assessments in occupational settings.

Respirable dust (RD) is an important occupational health hazard, particularly when it contains respirable crystalline silica. Exposure to silica-containing RD may cause silicosis, a progressive non-cancerous occupational lung disease. Although the U.S. Environmental Protection Agency (EPA) has not yet established a reference dose (RfD) or reference concentration (RfC) for quantitative assessment of the non-carcinogenic effects of RD containing crystalline silica, the cancer risk can be estimated using the Incremental Lifetime Cancer Risk (ILCR), a widely accepted measure that estimates the probability of developing cancer over a lifetime due to chronic exposure. ILCR values are calculated using either the average daily dose (ADD) or the lifetime average daily dose (LADD) combined with the cancer slope factor from inhalation (CSF), based on the linear, no-threshold assumption that even low levels of exposure may contribute to cancer risk [14]. Therefore, this study focused solely on carcinogenic outcomes by evaluating ILCR and did not attempt to quantify the risk of lung cancer or other non-cancerous respiratory diseases. Despite the well-established health risks associated with RD exposure, there remains a notable lack of probabilistic health risk assessment studies focusing on stone workers in Thailand. Most existing research has primarily concentrated on exposure assessment, disease prevalence, or health outcome evaluation, without integrating these findings into comprehensive quantitative risk models. For instance, Chaiyadej and Ketsakorn (2024) developed a rapid prediction model for silicosis risk among stone carvers in Nakhon Ratchasima, providing valuable insights into risk screening; however, the model did not incorporate probabilistic approaches to address variability and uncertainty in exposure parameters [8]. Similarly, Saiphoklang et al. investigated silicosis and pulmonary function among residents exposed to dust in Saraburi [15], while Silanun et al. reported the prevalence of silicosis among stone carving workers in Sikhio District, Nakhon Ratchasima [16]. Although these studies provide valuable epidemiological evidence and data on exposure, they do not quantify the potential lifetime cancer risk of RD exposure using standardized probabilistic risk assessment frameworks. Therefore, a critical research gap remains in the application of Monte Carlo simulation combined with ILCR estimation to characterize the uncertainty and variability of lifetime lung cancer risk among stone workers in Thailand.

## Materials and methods

### Study area and work processes

This study was conducted in Sikhio District, Nakhon Ratchasima Province, Thailand, a region recognized as a significant sandstone resource area. The sandstone deposits in this area were formed during the Mesozoic Era, specifically from the Late Jurassic to Early Cretaceous periods (approximately 140–65 million years ago), providing an abundant and readily accessible source of raw materials for stone processing and architectural carving [17]. As a result, stone carving has become a primary occupation and an important source of income for local communities. The study area is characterized by small-scale and informal production systems, where stone processing activities are typically performed in household-based settings. Stone processing and carving activities in the study area were classified into five main occupational categories based on task characteristics and exposure potential: mining, cutting, splitting, knocking, and stone carving, as described by Chaiyadej and Ketsakorn (2024) [8]. Mining involves sandstone extraction through mechanical and manual methods, generating high dust levels from drilling and material handling. Cutting uses electric saws to shape stone, producing substantial fine dust due to abrasion. Splitting breaks stone into smaller pieces using mechanical force, resulting in airborne dust during impact. Knocking involves surface refinement with hand tools, causing intermittent dust exposure. Stone carving, the final finishing process, entails continuous grinding and shaping, leading to prolonged exposure to fine dust and representing the highest risk for RD exposure.

### Study design and participations

This study employed a cross-sectional design to assess occupational exposure to RD and associated health risks among stone informal workers in Sikhio District, Nakhon Ratchasima Province, Thailand. The study focused on informal workers engaged in five main occupational categories, including mining, cutting, splitting, knocking, and stone carving. Eligible participants were individuals aged between 18 and 60 years who had been actively engaged in stone processing or carving activities for at least one year. A total of 243 participants were randomly selected for inclusion in the study. The total number of informal stone carvers in the study area was estimated to be approximately 310 individuals [18]. The required sample size was calculated using the Krejcie and Morgan (1970) formula [19] for a finite population with a 95% confidence level, a margin of error of 5%, and an estimated prevalence of 25.4% based on the study of Silanun et al. (2017) [20]. The calculated sample size was 242.88, which was rounded up to 243 participants. These participants were included in the questionnaire survey and collection of individual exposure-related information. For workplace exposure assessment, 83 personal air samples were collected according to the Similar Exposure Group (SEG) strategy to represent workers with comparable exposure characteristics rather than sampling every participant individually. Participants were selected using a proportionate stratified sampling method based on the distribution of workers across three subdistricts: Nongnamsai (n = 121), Khlong Phai (n = 147), and Khlong Tabaek (n = 42). A total sample size of 243 participants was obtained and proportionally allocated to each area, including Nongnamsai (n = 95), Khlong Phai (n = 115), and Khlong Tabaek (n = 33) [19]. Within each subdistrict, participants were recruited using purposive sampling to ensure representation of informal workers engaged in stone processing and carving activities.

### Data collection and instruments

Data collection was conducted using a combination of standardized questionnaires and personal air sampling to assess exposure to RD. The questionnaire was developed based on relevant literature and occupational health guidelines and consisted of three main sections: (1) demographic characteristics and work information (gender, age, marital status, education level, financial status, duration of employment in the area, weight, height, BMI, job functions, second job, working hour per day, working day per week, job experience, job before stone carving, workplace characteristics, and separation of residence from workplace), (2) basic health information, health behavior data, and prevention and control of occupational respiratory diseases (basic health information, working hours, duration of exposure), and (3) concentration of RD and exposure assessment. The content validity of the questionnaire was assessed using the Index of Item-Objective Congruence (IOC), and its reliability was tested prior to data collection. The questionnaire was administered through face-to-face interviews to ensure completeness and accuracy of responses.

RD concentrations in the workplace were measured using personal air sampling pumps (Universal PCXR8, SKC Inc., USA) equipped with 10-mm nylon cyclones (GS-1 Conductive Plastic Cyclone, SKC Inc., USA) and mixed cellulose ester (MCE) filters with a pore size of 0.8 µm (SKC Inc., USA). The sampling pumps were calibrated and operated at a flow rate of 1.7 L/min. Each sample was collected over a duration of 240 minutes in accordance with the National Institute for Occupational Safety and Health method [21], taking into account the irregular working patterns of stone carvers, who typically worked approximately 4 hours per day. The collected samples were analyzed for RD containing crystalline silica concentration using visible absorption spectrophotometry following NIOSH Method 7601 [21]. The analytical procedure involved the formation of silicon complexes, including silicomolybdate (measured at 420 nm) and molybdenum blue (measured at 820 nm). The limit of detection (LOD) for the analysis was 10 µg of SiO_2_. For samples with concentrations below the LOD, a substitution value equal to one-half of the LOD was applied.

RD containing crystalline silica samples collected on MCE filters were initially analyzed using Fourier Transform Infrared Spectroscopy (FT-IR) for quantitative determination of crystalline silica. The morphological characteristics of the collected particles, including particle size and particle shape were subsequently characterized using Scanning Electron Microscopy (SEM). SEM employs a focused electron beam to scan the sample surface and generate high-resolution three-dimensional images, enabling detailed observation of particle morphology. The morphology and particle size of the collected particles were examined using a JEOL JCM-6000 NeoScope benchtop scanning electron microscope (JEOL Ltd., Japan). Particle size was determined from calibrated SEM micrographs using the JCM-6000 SOFTWARE, Version 1.1. The distance-measurement function was used to measure particle dimensions, and particle size was reported as the maximum particle diameter (µm).

In addition to exposure assessment, the Incremental Lifetime Cancer Risk (ILCR) was evaluated to estimate the probability of cancer development associated with inhalation exposure to RD among stone informal workers. The ILCR was calculated based on the average daily dose (ADD) and the cancer slope factor (CSF), following the human health risk assessment framework recommended by the United States Environmental Protection Agency (U.S. EPA) [22]. The ADD for inhalation exposure was calculated using the following equation (1).


$$\begin{array}{c} ADD=\frac{C\times IR\times EF\times ED}{BW\times AT} \end{array}$$ (1)

where C is the concentration of RD in air (mg/m^3^), IR is the inhalation rate (m^3^/day), EF is the exposure frequency (days/year), ED is the exposure duration (years), BW is body weight (kg), and AT is the averaging time (days). For carcinogenic risk assessment, the averaging time was assumed to be equal to lifetime exposure (typically 70 years × 365 days).

The ILCR was then estimated using the following equation (2).


$$\begin{array}{c} ILCR=ADD\times CSF \end{array}$$ (2)

where CSF represents the cancer slope factor for inhalation exposure to RD containing crystalline silica. The cancer slope factor (CSF) applied in this study was derived from values reported by Goldsmith et al. [23]. The averaging time for carcinogenic risk was assumed to be equivalent to a lifetime exposure period. Default exposure parameters were obtained from established guidelines and relevant literature, while site-specific data were incorporated when available. The estimated ILCR values were interpreted based on the acceptable risk range recommended by the U.S. EPA, where a lifetime cancer risk between 1.0 × 10^−6^ and 1.0 × 10^−4^ is generally considered acceptable.

It should be noted that this risk assessment is limited only to the carcinogenic effects of RD. Although silicosis is the primary occupational disease associated with silica exposure, it is a non-carcinogenic outcome, and the U.S. EPA has not established a reference dose (RfD) or reference concentration (RfC) for silica exposure. Consequently, a quantitative non-cancer risk assessment was not performed using hazard quotient (HQ). Instead, the ILCR was estimated using an inhalation cancer slope factor derived from published epidemiological evidence, with the aim of assessing the potential lifetime risk of RD containing crystalline silica -related lung cancer.

To account for variability and uncertainty in exposure parameters, Monte Carlo simulation was performed using 10,000 iterations. Probability distributions were assigned to key input variables, including RD concentration, inhalation rate, and exposure duration. The simulation generated a distribution of ILCR values, from which descriptive statistics, including mean, median, and percentile values (5^th^, 50^th^ and 95^th^ percentiles), were derived.

### Data analysis

All data were analyzed using the Statistical Package for the Social Sciences (SPSS) software, version 27. Descriptive statistics were used to summarize demographic characteristics and work information, basic health information, health behavior data, and prevention and control of occupational respiratory diseases, and exposure levels of RD. Continuous variables were presented as means and standard deviations (SD), while categorical variables were expressed as frequencies and percentages. RD concentrations obtained from personal air sampling were analyzed to determine time-weighted average (TWA) exposure levels for each job functions. The measured concentrations were compared with the Thai occupational exposure limit (OEL) and the threshold limit value (TLV) to assess compliance with existing standards. The normality of continuous variables was assessed using the Shapiro–Wilk test. As the data were not normally distributed, non-parametric statistical methods were applied. Differences in RD concentrations and ILCR values among occupational groups were compared using the Kruskal–Wallis test. Statistical significance was determined at a p-value of less than 0.05.

The ILCR was calculated based on the ADD and CSF, following the U.S. EPA guidelines. To account for variability and uncertainty in exposure parameters, Monte Carlo simulation was performed with 10,000 iterations. Probability distributions were assigned to key input variables, including RD concentration, inhalation rate, exposure frequency, and exposure duration. The simulation outputs were used to estimate the distribution of ILCR values, including mean, median, standard deviation, and selected percentiles (5^th^, 50^th^ and 95^th^ percentiles).

### Ethical approval

Ethical approval for this study was granted by the Human Research Ethics Committee of Thammasat University, No.3 (Approval No. 061/2566), on July 24, 2023. Participant recruitment and data collection were conducted from August 1, 2023 to October 31, 2023. Prior to data collection, written informed consent was obtained from all participants. Participants were clearly informed about the study’s objectives, procedures, potential risks, and benefits. They were also assured of the confidentiality and anonymity of their responses and given the opportunity to ask questions before signing the consent form. All data were fully anonymized before being accessed by the research team. No minors were included in this study, and no waiver of consent was granted by the ethics committee.

## Results

### Participant characteristics

A total of 243 participants were included in the study, comprising 151 males (62.1%) and 92 females (37.9%). The majority of participants were aged 31–40 years and had an elementary level of education (66.0%). Most participants were married (78.2%) and reported sufficient financial status (46.5%). The most common job function was knocking (34.2%), followed by cutting (26.3%), splitting (16.8%), carving (12.8%), and mining (9.9%). Over half of the participants (58.4%) reported engaging in additional occupations, primarily farming. Most participants had more than 5 years of work experience (54.3%) and worked 8–12 hours per day. The majority did not have a second job (78.6%) and performed their work under both roofed and open-air conditions (74.9%). Additionally, more than half of the participants (54.7%) resided separately from their work areas, with an average distance of approximately 20 meters. Nearly half of the participants did not undergo annual health checkups. Most participants (91.8%) reported no underlying diseases requiring medical monitoring and primarily accessed healthcare services through the universal coverage scheme (69.1%). Approximately 60% had normal respiratory examination and chest X-ray results. Regarding health behaviors, 59.2% were nonsmokers, and about half reported washing their hands before meals, with eating areas generally separated from workspaces. Almost all participants reported showering after work, although the majority did not engage in regular exercise (81.9%). In terms of occupational health practices, 68.3% of participants reported receiving safety information on hazard prevention within the past year; however, 55.4% indicated that such information was received less than once per month. Despite this, 64.2% expressed willingness to share safety information with others. Dust control practices included spraying water at dust sources (61.7%) and around the workplace (69.3%). Nevertheless, the use of appropriate respiratory protective equipment was limited. Instead, participants commonly used improvised protection such as balaclava cloth coverings (48.2%), cotton masks (21.6%), and paper masks (16.1%), while only 14.1% reported using N95 respirators. Overall, participants demonstrated moderate levels of knowledge, attitudes, and practices regarding the surveillance, prevention, and control of occupational respiratory diseases.

The above results were obtained from prior peer-reviewed research conducted by Chaiyadej and Ketsakorn (2024) [8] and are incorporated in this study to contextualize and describe the demographic, occupational, and health characteristics of the study population.

### RD exposure levels

A total of 83 measurement points were used to assess respirable dust (RD) concentrations across five occupational categories. The results indicated that RD exposure varied by job function. The highest mean concentration was observed among stone carving workers (0.039 mg/m^3^), followed by splitting (0.035 mg/m^3^), cutting (0.025 mg/m^3^), knocking (0.022 mg/m^3^), and mining (0.006 mg/m^3^). The maximum RD concentration was also highest in the stone carving group (0.051 mg/m^3^), whereas the lowest concentrations were observed in mining, with some measurements below the limit of detection (LOD). Comparison with the applicable occupational exposure standards showed that the mean RD concentrations across the mining, cutting, splitting, knocking, and stone carving job functions were substantially lower than both the Thai occupational exposure limit (OEL)and the Occupational Safety and Health Administration (OSHA) permissible exposure limit (PEL), which were specified as 5 mg/m^3^. Although the mean RD concentrations were below the applicable occupational exposure limits, the stone carving and splitting job functions showed comparatively higher RD concentrations. These findings suggest a potential concern for respiratory health, especially in the presence of RD containing crystalline silica; however, the actual health risk should be interpreted in conjunction with the dust composition, exposure duration, and cumulative exposure. RD concentrations differed among job functions, as shown in Table 1.

**Table 1:** Workplace RD concentrations and particle characteristics.

Job functions	Sample size (n = 243)	Measurement points (n = 83)^a^	Workplace RD concentrations (mg/m³) and particle characteristics^b^				Standard (mg/m³)	
			Max	Min	Mean	SD	Thai OEL^d^	OSHA PEL^e^
**Mining**	24	14	0.012	<LOD^c^	0.006	0.006	5	5
**Cutting**	64	18	0.043	0.005	0.025	0.014		
**Splitting**	31	16	0.044	0.017	0.035	0.016		
**Knocking**	83	18	0.036	0.005	0.022	0.010		
**Stone carving**	41	17	0.051	0.028	0.039	0.012		

^a^Personal respirable dust (RD) air samples were collected from 83 representative workers selected using the Similar Exposure Group (SEG) strategy. The remaining participants contributed questionnaire and exposure parameter data but were not individually monitored for personal RD exposure. ^b^Representative SEM micrographs of dust particles collected on MCE filters are shown in Fig 1. The SEM observations demonstrated that the particles were predominantly irregular in shape, with particle diameters ranging from <1.3 to approximately 5.0 µm, indicating that the collected particles were within the respirable size fraction. ^c^LOD = Limit of Detection. ^d^Workplace concentrations of RD were assessed in comparison with the permissible exposure limit-time weighted average (PEL-TWA) of 5 mg/m^3^ set by the Department of Labour Protection and Welfare, Thailand. ^e^Workplace concentrations of RD were assessed in comparison with the permissible exposure limit-time weighted average (PEL-TWA) of 5 mg/m^3^ set by the Occupational Safety and Health Administration (OSHA)

Table 2 shows the comparison of time-weighted average (TWA) RD concentrations across different occupational groups was performed using the Kruskal-Wallis test. The results indicated a statistically significant difference in RD exposure among the groups (χ² = 50.363, p < 0.001). The mean rank values revealed that the stone carving group had the highest exposure level (mean rank = 69.12), followed by splitting (52.56), knocking (38.97), and cutting (34.42). In contrast, the mining group exhibited the lowest exposure level (mean rank = 10.64). These findings demonstrate a clear gradient of exposure intensity across job functions, with workers engaged in stone carving and splitting experiencing significantly higher RD concentrations compared to other occupational groups. The significant difference observed suggests that the type of work process plays a critical role in determining the level of respirable dust exposure.

**Table 2:** Comparison of TWA RD concentrations across occupational categories.

Job functions	Sample size (n)	Mean rank	Test statistic (χ²)	p-value
**Mining**	14	10.64	50.363	< 0.001
**Cutting**	18	34.42		
**Splitting**	16	52.56		
**Knocking**	18	38.97		
**Stone carving**	17	69.12		
**Total**	83	–		

### Probabilistic health risk assessment using Monte Carlo simulation

Given the variability in RD exposure and the uncertainty associated with individual and environmental parameters, a probabilistic approach was adopted for occupational health risk assessment. This approach facilitates the estimation of a full distribution of risk outcomes, rather than relying on deterministic point estimates, thereby offering a more robust and comprehensive evaluation of potential health risks. Monte Carlo simulation was conducted using input parameters described in Table 3, which were informed by direct field measurements and authoritative toxicological sources. Appropriate probability distributions (e.g., lognormal, triangular, and uniform) were assigned to each parameter to capture variability under real-world conditions. The outcomes of the Monte Carlo simulations, based on the defined input parameters and probability distributions for each job functions, are presented in Table 4 and Figs 2 and 3.

**Table 3:** Input parameters used in the Monte Carlo simulation for probabilistic human health risk assessment.

Parameter	Description	Unit	Values	Distribution	Source
**C**	RD concentration (TWA)	mg/m³	Measured values by job functions Mining: µ = 0.0092, σ = 0.0028 Cutting: µ = 0.0277, σ = 0.0087 Splitting: µ = 0.0275, σ = 0.0063 Knocking: µ = 0.0212, σ = 0.0052 Stone carving: µ = 0.0402, σ = 0.0042	Lognormal	This study
**IR**	Inhalation Rate	m^3^/day	Min = 15, Most Likely = 20, Max = 30	Triangular	[24]
**ET**	Exposure Time	hours/day	Mining: Min = 4, Most Likely = 7, Max = 8 Cutting: Min = 3, Most Likely = 8, Max = 12 Splitting: Min = 1, Most Likely = 7, Max = 9 Knocking: Min = 1, Most Likely = 8, Max = 12 Stone carving: Min = 5, Most Likely = 8, Max = 12	Triangular	This study
**EF**	Exposure Frequency	days/year	Mining: Min = 157, Most Likely = 320, Max = 365 Cutting: Min = 52, Most Likely = 316, Max = 365 Splitting: Min = 52, Most Likely = 324, Max = 365 Knocking: Min = 52, Most Likely = 324, Max = 365 Stone carving: Min = 261, Most Likely = 331, Max = 365	Triangular	This study
**ED**	Exposure Duration	years	Mining: Min = 1, Most Likely = 13, Max = 31 Cutting: Min = 1, Most Likely = 9, Max = 44 Splitting: Min = 1, Most Likely = 10, Max = 30 Knocking: Min = 1, Most Likely = 7, Max = 30 Stone carving: Min = 1, Most Likely = 9, Max = 40	Triangular	This study
**BW**	Body Weight	kg	Mining: µ = 59.9167, σ = 9.4865 Cutting: µ = 62.1094, σ = 10.0461 Splitting: µ = 61.8000, σ = 9.0446 Knocking: µ = 57.7973, σ = 11.4497 Stone carving: µ = 63.9512, σ = 9.7927	Lognormal	This study
**AT**	Averaging Time (lifetime)	days	Mining: Min = 8760, Max = 271,560 Cutting: Min = 8760, Max = 385,440 Splitting: Min = 8760, Max = 262,800 Knocking: Min = 8760, Max = 262,800 Stone carving: Min = 8760, Max = 350,400	Uniform	This study
**CSF**	Cancer Slope Factor	(mg/kg/day)^−1^	Min = 0.000309, Most Likely = 0.00843, Max = 0.0116	Triangular	[23]

**Table 4:** Summary of ILCR derived from Monte Carlo simulation (n = 10,000).

Job functions	Min	Max	Mean	SD	5^th^ Percentile	50^th^ Percentile	95^th^ Percentile
**Mining**	7.0 × 10 ^−10^	1.14 × 10 ^−5^	1.11 × 10 ^−6^	1.06 × 10 ^−6^	6.0 × 10 ^−8^	8.24 × 10 ^−7^	3.18 × 10 ^−6^
**Cutting**	1.10 × 10 ^−9^	3.08 × 10 ^−5^	2.60 × 10 ^−6^	3.00 × 10 ^−6^	1.26 × 10 ^−7^	1.61 × 10 ^−6^	8.45 × 10 ^−6^
**Splitting**	1.49 × 10 ^−8^	2.04 × 10 ^−5^	2.52 × 10 ^−6^	2.41 × 10 ^−6^	1.61 × 10 ^−7^	1.88 × 10 ^−6^	7.30 × 10 ^−6^
**Knocking**	8.40 × 10 ^−9^	1.81 × 10 ^−5^	1.76 × 10 ^−6^	1.87 × 10 ^−6^	1.18 × 10 ^−7^	1.16 × 10 ^−6^	5.61 × 10 ^−6^
**Stone carving**	2.63 × 10 ^−8^	4.27 × 10 ^−5^	4.16 × 10 ^−6^	4.08 × 10 ^−6^	2.64 × 10 ^−7^	3.07 × 10 ^−6^	1.21 × 10 ^−5^

**Fig 1:**
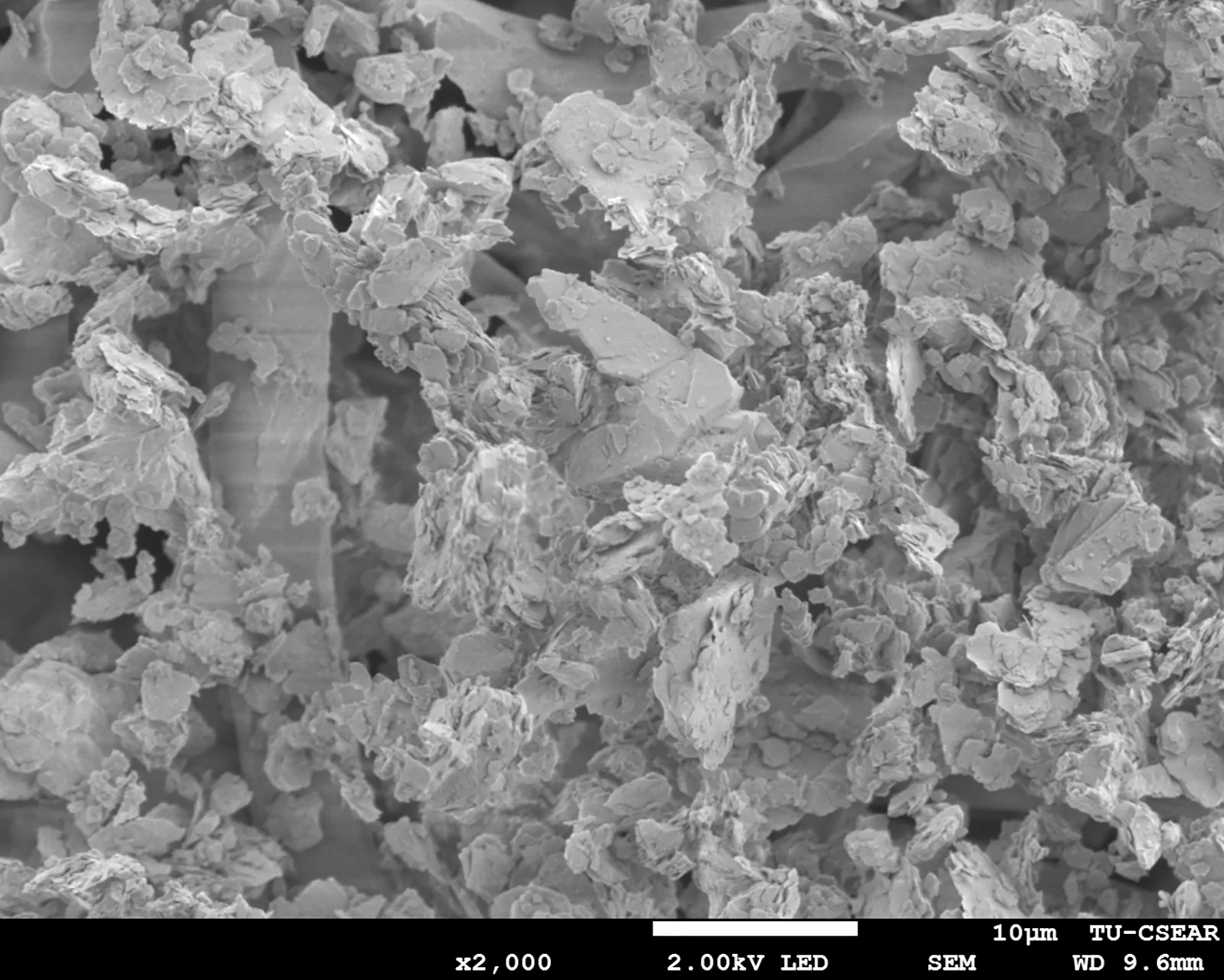
Representative SEM micrographs of dust particles.

**Fig 2:**
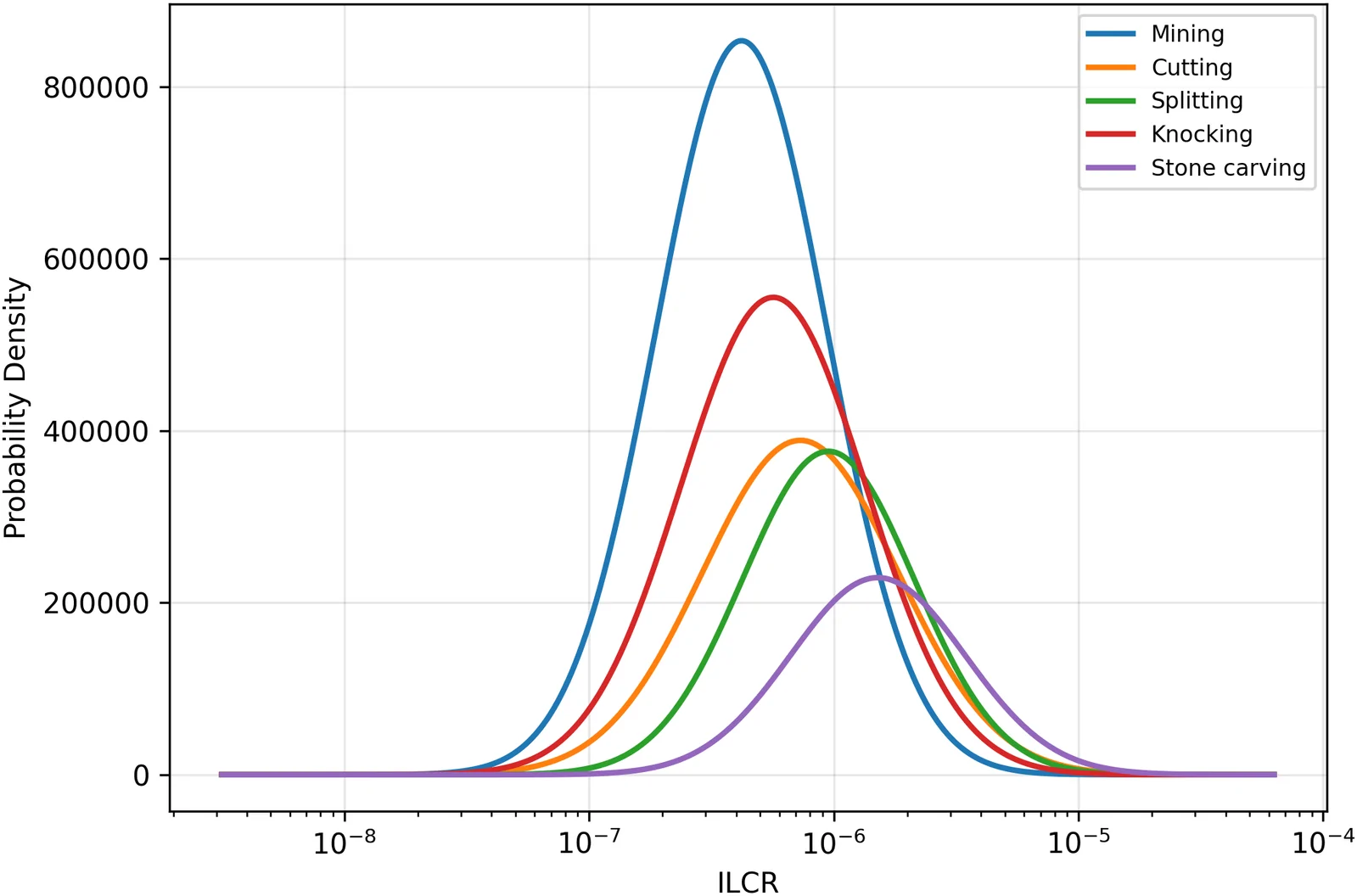
Probability density function (PDF) of ILCR.

**Fig 3:**
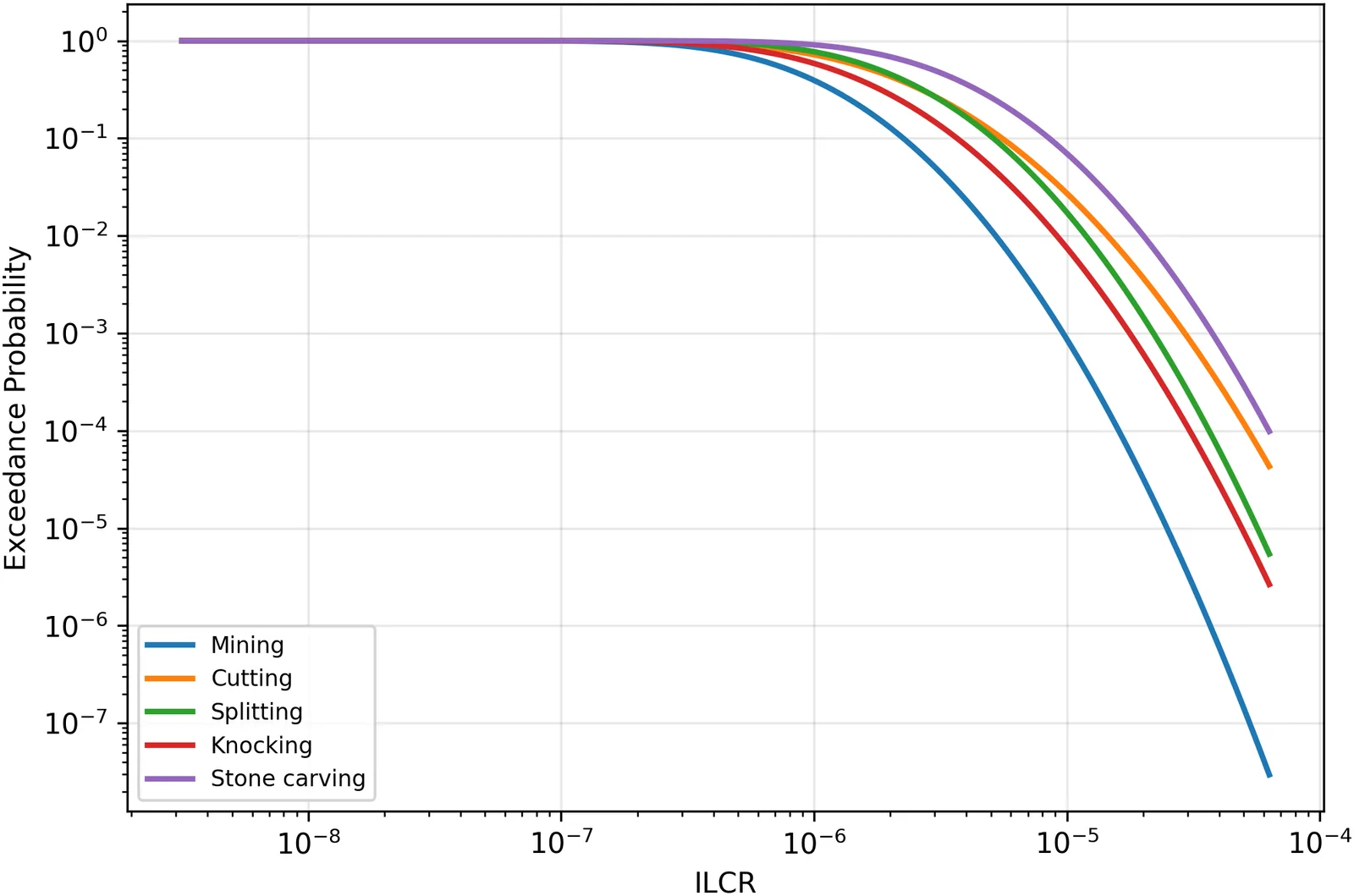
Complementary cumulative distribution function (CCDF) of ILCR.

Overall, the estimated ILCR values for all job functions fell within the risk management range of 1.0 × 10 ^−6^ to 1.0 × 10 ^−4^ generally considered acceptable by the U.S. EPA. However, differences in exposure intensity and variability were observed among job functions. Among all job functions, stone carving workers exhibited the highest estimated lifetime cancer risk, with a mean ILCR of 4.16 × 10 ^−6^ and a 95^th^ percentile of 1.21 × 10 ^−5^, suggesting that a subset of informal workers may experience elevated exposure levels. This group also showed the widest range of ILCR values (2.63 × 10 ^−8^ to 4.27 × 10 ^−5^), reflecting substantial variability in exposure conditions. The cutting and splitting groups demonstrated moderate risk levels, with mean ILCR values of 2.60 × 10 ^−6^ and 2.52 × 10 ^−6^, respectively. Their upper-bound estimates (P95) reached 8.45 × 10 ^−6^ and 7.30 × 10 ^−6^, indicating potential concern for high-exposure scenarios. Similarly, the knocking group showed a mean ILCR of 1.76 × 10 ^−6^, with a 95^th^ percentile of 5.61 × 10 ^−6^. In contrast, the mining group exhibited the lowest cancer risk, with a mean ILCR of 1.11 × 10 ^−6^ and a relatively lower upper-bound estimate (P95 = 3.18 × 10 ^−6^), suggesting comparatively lower exposure intensity. The distribution of ILCR values, particularly the differences between the 5^th^ and 95^th^ percentiles, highlights substantial uncertainty and variability in exposure, which were effectively captured using Monte Carlo simulation. The consistently higher upper-bound values (P95) compared to the means indicate that certain informal workers may experience significantly greater risks than the average. Sensitivity analysis suggests that ILCR variability is primarily driven by key exposure parameters, particularly RD concentration, followed by exposure duration and inhalation rate, reflecting differences in task characteristics and dust generation intensity across job functions.

The probability density functions (Fig 2) demonstrate right-skewed distributions for all job functions, indicating variability in exposure levels, with stone carving workers exhibiting the highest and most dispersed risk profile. Consistently, the complementary cumulative distribution functions (Fig 3) illustrate the probability of exceeding specific ILCR thresholds, showing that stone carving workers have the greatest exceedance probability, followed by the cutting and splitting groups, while mining workers display the lowest likelihood of elevated cancer risk.

## Discussion

This study employed a probabilistic approach using Monte Carlo simulation to assess the health risks associated with RD exposure among informal stone workers in Thailand. The results indicated that the estimated incremental lifetime cancer risk (ILCR) values across all job functions were within the acceptable risk range (10 ^−6^–10 ^−4^) recommended by the United States Environmental Protection Agency, suggesting that the overall cancer risk may be considered acceptable. However, the elevated upper-bound estimates (P95) observed in certain groups indicate that specific subpopulations of workers may still be exposed to higher levels of risk, underscoring the importance of incorporating variability and uncertainty into occupational health risk assessments. These findings are consistent with evidence from Thailand, where occupational exposure to stone dust has been associated with adverse respiratory health outcomes. Silanun et al. (2017) [16] reported a high prevalence of silicosis (36.1%) among stone workers in Sikhio District, Nakhon Ratchasima Province, particularly among those engaged in dust-intensive tasks such as stone cutting. Collectively, these findings reinforce the association between dust-generating activities and increased occupational health risks. Among the job functions examined, stone carving workers exhibited the highest ILCR values, followed by the cutting and splitting groups, whereas mining workers demonstrated the lowest risk levels. These findings are consistent with the characteristics of the work processes, as stone carving involves prolonged and intensive grinding and shaping activities that generate high concentrations of fine respirable dust [8,25]. Previous studies have similarly reported elevated exposure levels and increased health risks among workers engaged in dust-intensive tasks, particularly in informal or small-scale industries where engineering controls are often inadequate [7]. For example, Chaiyadej and Ketsakorn (2024) [8] identified stone carving as a high-risk occupation for silicosis among workers in Nakhon Ratchasima, Thailand. Similarly, Ketsakorn and Chaiyadej (2026) [25] developed a web-based predictive model for silicosis risk using multiple regression analysis, which classified 77.1% of participants as “very high risk.”

The scanning electron microscopy (SEM) observations further supported the exposure assessment by demonstrating that the collected dust particles were predominantly irregular in shape, with geometric diameters ranging from less than 1.3 to approximately 5.0 µm, which are generally consistent with the respirable particle size fraction capable of penetrating into the lower respiratory tract. Although mineralogical characterization was not performed on the samples collected in this study, it should be noted that SEM provides information on particle morphology and size but cannot distinguish between crystalline and amorphous silica. Therefore, the mineralogical phases of the collected particles could not be determined using SEM alone. Previous research conducted in a comparable sandstone-carving industry in Thailand by Kanala Chanvirat et al. (2018) used X-ray diffraction (XRD) and reported quartz (SiO_2_) contents ranging from 0.15% to 71.4% across different stone-processing job functions, demonstrating substantial variability in crystalline silica content according to work processes [26]. These published findings provide contextual evidence supporting the likelihood of crystalline silica exposure in similar occupational environments but should not be interpreted as representing the mineralogical composition of the samples analyzed in the present study. Nevertheless, the combination of Fourier Transform Infrared Spectroscopy (FT-IR) based quantification of RD containing crystalline silica and SEM characterization of respirable particle morphology provides complementary evidence supporting the exposure assessment and the biological plausibility of the associated long-term respiratory health risks among informal stone workers. The probabilistic analysis demonstrated substantial variability in ILCR distributions across job functions, as evidenced by wide percentile ranges and relatively high standard deviations. The right-skewed distributions observed in the PDF and CCDF plots indicate that a proportion of informal workers may be exposed to significantly higher levels of RD than the average. This finding aligns with previous research emphasizing that deterministic risk assessment methods may underestimate risk by failing to capture variability in exposure conditions and individual susceptibility [9]. In contrast, Monte Carlo simulation provides a more comprehensive framework by incorporating uncertainty and generating a range of possible outcomes, thereby improving the robustness of risk estimates [10,27]. Furthermore, recent advances in enhanced Monte Carlo methods have emphasized the importance of optimizing uncertainty estimation to improve the accuracy and interpretability of probabilistic analyses [13,28]. Sensitivity analysis indicates that RD concentration is the primary determinant of ILCR, followed by exposure duration and inhalation rate. This is consistent with established risk assessment frameworks, in which contaminant concentration directly influences the average daily dose (ADD) and subsequent cancer risk (US EPA) [29]. The observed variability across job functions can therefore be attributed to differences in dust generation intensity, work practices, and the effectiveness of exposure control measures. These findings are supported by previous studies; for example, Yeheyis et al. (2012) demonstrated the significance of variability in silica exposure among construction workers using a probabilistic risk analysis approach [30]. Similarly, Saeedizadeh et al. (2026) applied Monte Carlo simulation to assess occupational exposure to respirable crystalline silica among ceramic workers and highlighted the critical role of exposure parameters in determining health risk outcomes [31]. In addition, Mofidi et al. (2020) emphasized that variability in silica dust exposure and the effectiveness of control interventions significantly influence risk estimates and the evaluation of occupational health strategies [32].

Although this study used ILCR to assess the potential cancer risk from exposure to RD, these estimates should not be interpreted as an indicator of silicosis risk. Silicosis remains the most common occupational disease associated with crystalline silica exposure and should be assessed through exposure monitoring, clinical surveillance, pulmonary function tests, and radiological examinations, rather than cancer risk models. Therefore, ILCR results should be considered complementary to conventional workplace health surveillance, providing additional information on the potential long-term cancer-causing effects of chronic silica exposure. From a practical perspective, these findings highlight the need for targeted occupational health interventions in informal stone processing settings in Sikhio District, Nakhon Ratchasima Province. Engineering controls should emphasize low-cost measures, such as water spraying at dust sources and simple local exhaust ventilation (LEV) in workshop settings, while home-based workers should be encouraged to use portable water sprays and work in well-ventilated areas. The use of appropriate respiratory protection, particularly N95 respirators, should replace inadequate face coverings. In addition, community-based training and education programs should be implemented to improve awareness of respirable dust hazards and safe work practices. Periodic health surveillance, including chest radiography and pulmonary function testing when clinically indicated, should be strengthened through local healthcare services to support the early identification of silicosis and other occupational respiratory conditions associated with respirable dust exposure. Given the informal nature of the workforce, enhanced local enforcement, outreach activities, and the integrating digital tools, such as the web-based silicosis risk assessment model developed in previous studies, may further support risk communication and encourage behavioral change among workers.

## Conclusions

This study demonstrates the value of probabilistic risk assessment in capturing variability and uncertainty in RD exposure among informal workers. Unlike deterministic approaches, this method provides a more realistic representation of exposure and risk, highlighting that reliance on average estimates may overlook vulnerable subgroups. The findings underscore the importance of considering upper-bound risks in occupational health decision-making, particularly for dust-intensive tasks. From a practical perspective, targeted and context-appropriate interventions are essential, including low-cost engineering controls, improved respiratory protection, worker education, and strengthened health surveillance. In addition, the integration of a web-based risk assessment tool offers a practical approach to enhance risk communication, support real-time risk evaluation, and promote preventive behaviors among workers. Overall, probabilistic approaches combined with digital tools provide a robust decision-support framework for prioritizing interventions and informing policies aimed at reducing respirable dust-related health risks in informal sectors.
